# HLS_19_-NAV—Validation of a New Instrument Measuring Navigational Health Literacy in Eight European Countries

**DOI:** 10.3390/ijerph192113863

**Published:** 2022-10-25

**Authors:** Lennert Griese, Hanne S. Finbråten, Rita Francisco, Saskia M. De Gani, Robert Griebler, Øystein Guttersrud, Rebecca Jaks, Christopher Le, Thomas Link, Andreia Silva da Costa, Miguel Telo de Arriaga, Rajae Touzani, Mitja Vrdelja, Jürgen M. Pelikan, Doris Schaeffer

**Affiliations:** 1 School of Public Health, Bielefeld University, 33615 Bielefeld, Germany; 2 Department of Health and Nursing Sciences, Faculty of Social and Health Sciences, Inland Norway University of Applied Sciences, 2418 Elverum, Norway; 3 Católica Research Centre for Psychological, Family and Social Well-Being, Universidade Católica Portuguesa, 1649-023 Lisboa, Portugal; 4 Careum Foundation, Careum Center for Health Literacy, 8032 Zurich, Switzerland; 5 Careum School of Health, Kalaidos University of Applied Sciences, 8006 Zurich, Switzerland; 6 Competence Centre for Health Promotion and Health System, Austrian National Public Health Institute, A-1010 Vienna, Austria; 7 Department of Nursing and Health Promotion, Faculty of Health Sciences, Oslo Metropolitan University, 0130 Oslo, Norway; 8 Department of Social Determinants of Health, Division of Prevention and Public Health, Norwegian Directorate of Health, 0213 Oslo, Norway; 9 Department of Quality Measurement and Patient Survey, Austrian National Public Health Institute, A-1010 Vienna, Austria; 10 Nursing Research, Innovation and Development Centre of Lisbon (CIDNUR), Nursing School of Lisbon (ESEL), 1049-005 Lisboa, Portugal; 11 Instituto de Saúde Ambiental (ISAMB), Faculdade de Medicina, Universidade de Lisboa, 1649-026 Lisboa, Portugal; 12 Direção-Geral da Saúde, 1049-005 Lisboa, Portugal; 13 Aix Marseille Univ, INSERM, IRD, ISSPAM, SESSTIM, Sciences Economiques & Sociales de la Santé & Traitement de l’Information Médicale, Equipe CANBIOS Labellisée Ligue 2019, 13009 Marseille, France; 14 Institut Paoli-Calmettes, SESSTIM U1252, 13009 Marseille, France; 15 Communication Unit, National Institute of Public Health, 1000 Ljubljana, Slovenia; 16 WHO-CC Health Promotion in Hospitals and Health Care, Austrian National Public Health Institute, A-1010 Vienna, Austria

**Keywords:** health literacy, health information, navigation, health care system, confirmatory factor analysis, instrument, questionnaire, HLS_19_ survey, Rasch modelling, validation

## Abstract

To manoeuvre a complex and fragmented health care system, people need sufficient navigational health literacy (NAV-HL). The objective of this study was to validate the HLS_19_-NAV measurement scale applied in the European Health Literacy Population Survey 2019–2021 (HLS_19_). From December 2019 to January 2021, data on NAV-HL was collected in eight European countries. The HLS_19_-NAV was translated into seven languages and successfully applied in and validated for eight countries, where language and survey method differed. The psychometric properties of the scale were assessed using confirmatory factor analysis (CFA) and Rasch modelling. The tested CFA models sufficiently well described the observed correlation structures. In most countries, the NAV-HL data displayed acceptable fit to the unidimensional Rasch partial credit model (PCM). For some countries, some items showed poor data–model fit when tested against the PCM, and some items displayed differential item functioning for selected person factors. The HLS_19_-NAV demonstrated high internal consistency. To ensure content validity, the HLS_19_-NAV was developed based on a conceptual framework. As an estimate of discriminant validity, the Pearson correlations between the NAV-HL and general health literacy (GEN-HL) scales were computed. Concurrent predictive validity was estimated by testing whether the HLS_19_-NAV, like general HL measures, follows a social gradient and whether it forms a predictor of general health status as a health-related outcome of general HL. In some countries, adjustments at the item level may be beneficial.

## 1. Introduction

The ability to navigate the health care system (HCS) is increasingly important owing to more complex HCSs with their various sectors and myriad of organizations [1,2,3,4,5,6,7]. Complexity and fragmentation of HCSs lead to a lack of transparency and increased demands on patients and users to access the right care, at the right time, at the right place [8,9,10,11,12,13,14]. Navigating the HCS is also challenging because patients and users are increasingly expected to take responsibility for their own health and healthcare and thus face the challenge of independently gathering and managing health information from a wide range of services and information sources [15,16]. However, numerous patients and users lack the required skills and information [17,18,19,20]. As a result, they may endure a tiring odyssey through the maze of the HCS, getting lost, experiencing dead ends, and receiving delays in diagnosis or treatment, which is also related to low satisfaction and even trust in the HCS and health professionals [21,22,23].

To avoid such consequences, navigating the HCS requires health literacy (HL), or more specifically, navigational health literacy (NAV-HL), which is defined as *“people’s knowledge, motivation, and skills to access, understand, appraise, and apply the information and communication in various forms necessary for navigating health care systems and services adequately to get the most suitable health care for oneself or related persons”* [24] (p. 6). In particular, this applies to frequent users of the HCS and their significant others, especially to people with chronic illness who naturally have more health care needs; needs that change frequently, become more complex over time, and which may include multiple layers of health care [25,26,27,28,29]. In consequence, they are particularly exposed to the HCS and must continually acquaint themselves with new health care settings and services, making them especially reliant on adequate NAV-HL [30]. NAV-HL may also matter since low HL has been identified as a barrier for understanding and using the HCS, as supporting an inappropriate use of health care services, and as a cause of potentially higher health expenditure [31,32].

However, following the relational model of HL—describing HL as the result of individual skills and abilities, but also of the demands and complexity individuals face in dealing with health information [33]—the low availability and comprehensibility of navigation-related information led to the hypothesis of poorly developed NAV-HL in general populations. Navigation-related information usually meets criteria relevant to organizations within the HCS but does not sufficiently consider challenges faced by its users [30,34,35], i.e., it is characterized by low user orientation and usability.

Nevertheless, as has been pointed out elsewhere [17,24], so far, little is known about the NAV-HL in general populations. In HL research, HCS navigation has received infrequent or irregular attention. Only a few published studies exist. For example, in an exploratory study of hospital navigability, Rudd [36] referred to hospitals as complex “literate environments” in which it is difficult for users to find the right point of contact. In another study, Rudd et al. [37] (p. 8) analysed literacy tasks regarding “rights and responsibilities, application for insurance and other coverage plans, and informed consent for procedures and studies” and labelled “systems navigation” as one of five domains of the Health Activities Literacy Scale (HALS) [37,38,39]. While these latter studies mainly emphasis the functional domain of HL, Osborne et al. [40] operationalized HCS navigation as one of nine subscales of their Health Literacy Questionnaire (HLQ). However, the HLQ navigation dimension reflects only to a limited extent the comprehensive definition of HL of Sørensen et al. [17,24,30,41].

Apart from this small number of existing studies, there are no studies introducing instruments on HL in the specific field of navigating the HCS [17]. For this reason, a new instrument measuring NAV-HL, the HLS_19_-NAV, was developed as part of the European Health Literacy Population Survey 2019–2021 (HLS_19_) [17,24]. The HLS_19_-NAV was applied for the first time in HLS_19_. Data were collected on the scale in eight countries (Austria (AT), Belgium (BE), Czech Republic (CZ), France (FR), Germany (DE), Portugal (PT), Slovenia (SI), Switzerland (CH)) by using different methods of survey data collection.

This article is part of a series of already published [42] and upcoming papers introducing new HL tools that have been developed, applied, and tested, all in the HLS_19_ study. In general, the aim of these articles is to use the data collected in HLS_19_ to examine the psychometric properties of the newly developed HL tools and different aspects of its validity. To derive overarching and comparable conclusions about the HLS_19_ tools, the single papers address similar research questions, and use partly the same data and analyses procedures. For this article, it is asked:

1. How well does a single-factor, as compared to a two-factor confirmatory factor analysis (CFA) model, describe the correlation structure of the HLS19-NAV data?
2. To which extent does the data fit the unidimensional Rasch model?
3. What is the impact of using dichotomous or polytomous data on the psychometric properties of the NAV-HL scale?
4. How well does the instrument fulfil aspects of content and face validity and of construct validity measured as discriminant and concurrent predictive validity?

For answering these research questions, part of the analyses based on dichotomous data of the HLS_19_-NAV scale from the respective chapter in the “International Report on the Methodology, Results, and Recommendations” [17] are presented and supplemented by new additional analyses based on polytomous data.

## 2. Materials and Methods

### 2.1. Development of the HLS_19_-NAV

The working group for the development of the HLS_19_-NAV was mainly led by German researchers as part of the German national health literacy survey (HLS-GER 2) with which Germany participated in HLS_19_ [43]. Prior to developing the HLS_19_-NAV, a scoping review of definitions, concepts, and tools related to HCS navigation was conducted. From the literature, three aspects were identified: Information tasks relevant for navigating the HCS at the system level (how the HCS is organized, how it functions and works), at the organizational level (how to choose a suitable health care organization, how to use it and find one’s way), and at the interactional level (how to interact with health professionals and organizations to negotiate health care paths and settings). Initially, 15 items were developed and tested in an expert review (*n* = 6). Item comprehensibility and interpretation were evaluated by using four focus group discussions, each with eight participants, and later by using cognitive interviews (*n* = 33). Further details are available in Griese et al. [24]. The 12 items selected for the final measurement scale (Table 1) reflect the four cognitive operations access (3 items), understand (3 items), appraise (3 items), and apply (3 items), for information on navigational issues. Polytomous responses were collected by using a four-point rating scale (4 “very easy”, 3 “easy”, 2 “difficult”, 1 “very difficult”).

### 2.2. Translation Procedure

For this study, the HLS_19_-NAV was translated into seven languages (Czech, Dutch, French, German, Italian, Portuguese, and Slovenian). Two forward translations were performed in SI and BE (Dutch version). This was done by the responsible study team and by a national data collection agency. Countries with common languages collaborated in the translation process: In AT, CH, and DE, one forward translation for the German version was conducted by the national researchers and one by the German national data collection agency. The two versions were compared, and consensus was reached within the AT, CH, and DE study teams. The German version was translated into French and Italian by the language service of the Swiss Federal Office of Public Health (FOPH), reviewed by different experts, and consented on between the BE, FR, and IT study teams. One forward translation was conducted in CZ and PT. Additional backward translations were performed by the CZ and SI teams [17].

### 2.3. Data Collection

The HLS_19_ survey collected cross-sectional data in 17 countries within the wider WHO European Region (HLS_19_ Consortium 2021), where 8 countries (Table 2) collected data on all 12 items of the optional HLS_19_-NAV scale. Four survey methods were available: Computer-assisted personal interviewing (CAPI), pen-and-paper personal interviewing (PAPI), computer-assisted telephone interviewing (CATI), and computer-assisted web interviewing (CAWI). Three countries used a combination of methods (Table 2). Most countries used a multi-stage random sampling or quota sampling procedure (Table 2). The total sample size was smallest in BE, with *n* = 1000 (BE), and largest in SI, with *n* = 3360 (Table 2) [17].

### 2.4. Other Variables Included in the Analysis

Among sociodemographic and socioeconomic variables, gender, age (in years), self-assessed social status (from 1 “lowest self-assessed social status” to 10 “highest self-assessed social status”) [44], the highest level of completed education (lower secondary education or below: ISCED 0–2; higher secondary education: ISCED 3; above secondary education: ISCED 4–8) [45,46], and a self-assessment item on difficulties in “paying all bills at the end of the month” (four response categories from 4 “very easy” to 1 “very difficult”) were included to describe sample characteristics. Additionally, data on respondent employment status (employed and unemployed or retired), self-reported general health status ((very) good or fair and (very) bad) were used to test for differential item functioning (DIF). For DIF analyses, variables on education (ISCED 0–3 and 4–8) and social status (levels 1–4 and levels 5–10) were dichotomized and various age categories were computed [47]. Variables entered in the regression model include the NAV-HL score (0–100), age, education (ISCED 0–8), self-assessed social status (1–10), financial deprivation (4 categories, from no deprivation (0) to severe deprivation (100)) and self-reported general health status [17].

General health literacy (GEN-HL) was measured using the HLS_19_-Q12 self-assessment scale, which is a 12-item revised short form of the HLS-EU-Q47 [17,48]. The HLS_19_-Q12 captures a comprehensive and public health-oriented concept of HL by operationalizing a conceptual framework of three health domains (health care, disease prevention, health promotion) combined with the previously mentioned cognitive operations (to access, understand, appraise, and apply health information). Using the same four-point rating scale as the HLS_19_-NAV (4 “very easy”, 3 “easy”, 2 “difficult”, 1 “very difficult”), the HLS_19_-Q12 measures perceived difficulties in accomplishing HL tasks.

### 2.5. Analysis

The HLS_19_ study is based on the HLS-EU study from 2012 [18], which proposed a standardized sum score using polytomous responses (“very easy”, “fairly easy”, “fairly difficult”, “very difficult”). However, HLS_19_ scale scores were calculated using dichotomized or rescored data (the “very easy”/“easy” and “very difficult”/“difficult” responses were combined, respectively). Since the initial results reported in the International Report of HLS_19_ [17] were based on rescored or dichotomized data, one aim of this article is to explore how rescored data affect the psychometric properties of the HLS_19_-NAV compared to using the original polytomous responses. Analyses on psychometric properties are based on CFA and Rasch modelling.

Concerning CFA, a factor model reflecting the NAV-HL framework was computed. The NAV-HL framework [24] describes three domains or levels referred to as the organizational level (items HLS_19_-NAV1-5), system level (items HLS_19_-NAV6-11) and interactional level (item HLS_19_-NAV12). Discarding item HLS_19_-NAV12, which represents the interactional level, a two-factor CFA model was fitted on each sample. Single-factor CFA models, where HLS_19_-NAV12 was included, were additionally estimated, as HLS_19_ reported on a single overall score for all 12 HLS_19_-NAV scale items [17]. Owing to categorical data, we used the lavaan package for R [49] with a diagonally weighted least-squares estimator (DWLS) [50,51,52]. A good or sufficient model fit was assumed if the following target values of the applied goodness-of-fit indices were met: standardized root-mean-squared residual (SRMR ≤ 0.08), root-mean-squared error of approximation (RMSEA ≤ 0.06), comparative fit index (CFI ≥ 0.95), Tucker–Lewis index (TLI ≥ 0.95), goodness-of-fit index (GFI ≥ 0.95), and adjusted goodness-of-fit index (AGFI ≥ 0.9) [50,53,54]. Due to the large sample sizes, no chi-squared values are reported. Standardized parameter estimates, or rather the respective R^2^ values, were examined for low values. The residual correlation matrix was inspected for coefficient values greater than 0.1 as possible indicators of a possible model–data disagreement [51].

RUMM2030Plus [55] and ACER ConQuest 5 [56] were used for Rasch modelling [47]. Data were tested against the partial credit parameterization (PCM) of the unidimensional Rasch model [57]. Overall data–model fit was assessed by chi-square fit statistics, and scale targeting was evaluated by comparing the distribution of item locations to the distribution of person locations. Several tests of unidimensionality are available. Using dependent *t*-tests [58], we reported the proportion of respondents with significantly different location estimates based on the system level subscale (items HLS_19_-NAV1-5) and the organizational level subscale, including the item measuring the interactional level (items HLS_19_-NAV6-12). Comparing score on two theoretically defined subscales, we assume strict unidimensionality when less than 5% of *t*-tests are significant. However, constructing scales by a composition of subscales to increase validity, some multidimensionality is inevitable.

At the item level, single-item fit, differential item functioning (DIF), response dependency, and ordering of response categories were evaluated [47].

Single-item chi-square probability values above a Bonferroni-adjusted 5% level, fit residuals within the range of ±2.5, and Infit values between 0.7–1.3 indicate sufficient item fit [59,60,61]. Differential item functioning (DIF) refers to differences in item performance between the respondent groups we match with respect to the construct we measure. We matched groups on gender, age, education, status of employment, financial deprivation, social level, and/or general health status. We refer to uniform DIF when items have different relative difficulty and non-uniform DIF when items discriminate differently for different groups of people. An overview of person factor categories is available in Table S1 of the Supplementary file. DIF was evaluated by using two-way analysis of variance [62] and inspecting graphical displays. We used a Bonferroni-adjusted significant probability value of <5%.

Since models tested on large data sets run the risk of being rejected due to the chi-squared statistic [63], analyses on data–model fit, item fit, and DIF were based on reduced sample sizes. Andrich and Marais [62] recommend using a sample size corresponding to 10–30 persons per threshold, where the total number of thresholds equals the product of the number of items (12) and the number of thresholds per item (3), yielding a sample size between *n* = 1080 and 360 when assessing the psychometric properties of the HLS_19_-NAV. By “ordered response categories”, we mean significantly different item thresholds that are in “correct” order [62].

The items should only be correlated through the latent trait being measured. Using Rasch modelling, a residual correlation refers to the relationship between two items whilst taking away the effects of the latent variable on this relationship. A residual correlation between items of >0.3 was applied to detect response dependency [64].

As a measure of internal consistency, Cronbach’s alpha and Omega for categorical data were estimated. The Person Separation Index (PSI) was computed to estimate the lower limit of the true reliability. In general, reliability indices refer to how well a scale is able to separate between respondents along the latent trait. The reliability was considered acceptable when indices exceeded 0.7 [51]. In addition, the average variance extracted (AVE) was calculated and interpreted using a limiting value of AVE ≥ 0.5 [65].

To ensure content or face validity, the HLS_19_-NAV was developed and tested, as mentioned above, based on the conceptual NAV-HL framework, which defines three levels relevant for navigating the HCS while referring to the multidimensional HL definition of Sørensen et al. [24,41].

As part of construct validity, discriminant validity, meaning that two theoretically different measures should not be too highly related [66], was tested by Pearson correlations between the NAV-HL and GEN-HL scores. Scores were standardized to the range of 0 to 100 [17]. A higher score indicates higher NAV-HL/GEN-HL (distributions of NAV-HL scores based on dichotomous and polytomous scoring can be found in the Supplementary file: Figures S1 and S2). As the NAV-HL and GEN-HL scales are based on the same HL construct [41], it was hypothesized that the measures would correlate to a certain degree.

Furthermore, it was tested (a) whether NAV-HL is determined by factors that were already identified as indicators for the presence of a social gradient in HL research and (b) whether NAV-HL predicts health-related consequences, here general health status, that were already found to be associated with general HL [18]. For (a), linear regression models, including NAV-HL score as dependent variable and gender, age, education, self-assessed social status, and financial deprivation as hypothesized predictor variables, were calculated. For (b), linear regression analyses were performed including the NAV-HL score and the mentioned social variables as predictor variables of general health status. In this regard, the aim was not to examine an optimal model that best explains the dependent variable, but to examine whether NAV-HL is related to the considered factors as expected. As sample sizes in CH (CATI: *n* = 192) and CZ (CATI: *n* = 532) were considerably smaller compared to other countries, in this case, data from the total country samples were used.

## 3. Results

The sample characteristics are presented in Table 3. In total, valid data on NAV-HL were collected for an overall of 15,685 respondents across the eight countries. In most countries, the number of missing values (cases not used to calculate the score because less than 80% of HLS_19_ NAV items were answered) was small, varying between 0% and 2%. In AT (5%), CH (10% for CATI), CZ (10% for CATI), and PT (14%), missing values were higher.

### 3.1. Confirmatory Factor Analysis

Fitting the single-factor CFA (Table 4) to dichotomous data, most goodness-of-fit indices indicated good to sufficient fit for most countries. SRMR varied between the acceptable values 0.03 (CZ, CAWI) and 0.07 (CH, CAWI, and DE), while the RMSEA of 0.07 observed for BE, CH (CAWI), and PT was slightly above the strict target value ≤0.06 [67]. With a minimum of 0.97, the observed values for the CFI, TLI, GFI, and AGFI are sufficient. The CFA analysis based on polytomous data points to similar results. The fit indices for the model remained stable except for the RMSEA, where values were considerably higher for polytomous data.

For the dichotomous data, the standardized parameter estimates are above 0.7 for all items except for the German data and for item HLS_19_-NAV9 (to understand how to get an appointment with a particular health service) in AT, BE, CH, and FR. The CFA for the polytomous data shows comparable results with only minor differences in the range of (−0.05 to 0.06). Except for the German data, the R^2^ value is above 0.5 for all items but HLS_19_-NAV9, with the R^2^ values being minimally higher on average (by 0.02) for the dichotomous data.

For all country-specific samples, there were entries >0.10 [68] in the residual correlation matrix. Of the 66 possible residual correlation coefficients (cf. Tables S2 and S3), on average, 6 values are above 0.1. For the dichotomous data, the number of residual correlation coefficients above 0.1 is the highest for CH (CAWI: 12 times), DE (11 times), BE (8 times), CH (CATI: 7 times), and PT (7 times). The residual correlation coefficients are above 0.1 for the majority of data sets for HLS_19_-NAV1 with HLS_19_-NAV2. The residual correlation coefficients of HLS_19_-NAV1 or HLS_19_-NAV2 with HLS_19_-NAV7 or HLS_19_-NAV8 could also require further inspection. For the polytomous data, the data sets of BE, CH (CAWI, CATI), and DE show 10 or more reidual correlation coefficients that are higher than 0.1. The variables concerned are the same as for the dichotomous data. All residual correlation coefficients are below 0.2 with the exception of the residual correlation of HLS_19_-NAV11 with HLS_19_-NAV10 for the Swiss (CATI) polytomous data (r_res_ = 0.2).

Fitting the two-factor CFA model (Table 5) to dichotomous data, fit indices improved compared to the single-factor CFA, with lower SRMR values and/or RMSEA values in AT (SRMR = 0.04; RMSEA = 0.04), BE (SRMR = 0.05; RMSEA = 0.05), CH (SRMR = 0.06; RMSEA = 0.06 (CAWI); SRMR = 0.05; RMSEA = 0.00 (CATI)), CZ (SRMR = 0.03; RMSEA = 0.00 (CAWI); SRMR = 0.05; RMSEA = 0.00 (CATI)), DE (SRMR = 0.06; RMSEA = 0.06), FR (SRMR = 0.04; RMSEA = 0.05), PT (SRMR = 0.04; RMSEA = 0.04), and SI (SRMR = 0.04; RMSEA = 0.04 (CAWI); SRMR = 0.04; RMSEA = 0.03 (CAPI)). The values for the model fit coefficients CFI, TLI, GFI, and AGFI are also slightly higher (at most 0.01) for the two-factor model for some combinations of country and survey type. In the two-factor CFA models, the correlation coefficient between the two latent variables ranges from 0.84 to 0.96, which hints at the two factors being hardly distinguishable. The use of polytomous data results in lower SRMR in some countries, while the RMSEA, as in the single-factor model, increases.

### 3.2. Rasch Analyses at the Overall Level

When performing Rasch modelling based on dichotomous as opposed to polytomous data, it became evident, as expected, that the power of analyses of fit and reliability indices decreased and that the proportion of respondents with extreme scores increased (Table S4). Thus, it was decided to report results from Rasch modelling (with exception to PSI) based on polytomous HLS_19_-NAV data (taken from Guttersrud et al. [47]).

With a reduced sample size (*n* = 720: 20 persons per threshold), good overall data–model fit for the HLS_19_-NAV was observed in AT (χ^2^: *p* > 0.05), and sufficient overall data–model fit (χ^2^: *p* > 0.01) was observed in CH (CAWI, CATI), CZ (CAWI, CATI), and DE (Table 6). When sample size was further reduced (*n* = 360: 10 persons per threshold), data from BE, PT, and SI (CAWI, CAPI) also displayed sufficient to good data–model fit. The data collected in FR did not fit well to the PCM.

Within countries, the distribution of HLS_19_-NAV item threshold locations was well-targeted at the distribution of person locations, with mean person location varying between −0.31 (DE) and 0.96 (SI, CAWI).

Testing for dimensionality revealed that the HLS_19_-NAV scale is not strictly unidimensional: Using dependent *t*-tests, between 4.2% (CZ, CATI) and 12.2% (DE) of respondents obtained significantly different scores or proficiency estimates on the organizational level and system level subscales. Thus, too many respondents obtained too different subscales scores to claim that the two subscales measure the same trait.

### 3.3. Rasch Analyses at the Item Level

Based on reduced sample sizes of *n* = 1080 in each country (except BE (*n* = 1000) and CZ (CAWI *n* = 1067; CATI *n* = 532)), item HLS_19_-NAV9 (understand how to get an appointment with a particular health service) showed significant misfit and under-discriminated relative to the PCM in Belgian (*p* < 0.001, fit residual: 4.39, Infit: 1.39) and French (*p* < 0.001, fit residual: 8.60, Infit: 1.54) data. This was also valid when sample size was reduced to *n* = 720 in each country. Item HLS_19_-NAV9 also tended to under-discriminate relative to the PCM in Austrian (*p* < 0.001, fit residual: 6.01, Infit: 1.24) and Swiss (*p* < 0.001, fit residual: 2.71, Infit: 1.22 (CAWI); *p* < 0.001, fit residual: 1.55, Infit: 1.31 (CATI)) data. Furthermore, item HLS_19_-NAV12 (stand up for yourself if your health care does not meet your needs) showed significant misfit and under-discriminated in CZ (*p* < 0.001, fit residual: 8.14, Infit: 1.35) and SI (CAWI) (fit residual: 9.34, Infit: 1.44). Significant chi-square values and z-fit residuals outside the range of ±2.5, but acceptable Infit values, were observed for the following items: HLS_19_-NAV2 (judge which type of health service you need in case of a health problem) in FR (*p* < 0.001, fit residual: 2.95, Infit: 1.21) and in PT (*p* < 0.001, fit residual: −3.32, Infit: 1.02), for item HLS_19_-NAV4 (understand information on ongoing health care reforms that might affect your health care) in PT (*p* < 0.001, fit residual: −2.70, Infit: 1.05), for item HLS_19_-NAV8 (judge if a particular health service will meet your expectations and wishes on health care) in PT (*p* < 0.001, fit residual: −5.98, Infit: 0.84), for HLS_19_-NAV9 (understand how to get an appointment with a particular health service) in PT (*p* < 0.001, fit residual: −3.65, Infit: 1.09) and SI (CAPI) (*p* < 0.001, fit residual: −3.67, Infit: 1.02), and for HLS_19_-NAV10 (find out about support options that may help you to orientate yourself in the health care system) in DE (*p* < 0.001, fit residual: −5.58, Infit: 0.85). Results at the item level can be found in the supplementary file (Tables S5–S15, mainly taken from Guttersrud et al. [47]).

Several HLS_19_-NAV items displayed DIF when the sample size was set at *n* = 1080 (Tables S5–S15), but there was no pattern in which items displayed DIF for specific person factors across the countries. For some items, DIF was also evident when the sample size was reduced to *n* = 720. In FR and CH (CAWI), respondents aged 46 and older tended to score higher on this item compared to younger respondents despite the same level of NAV-HL. Item HLS_19_-NAV3 also displayed DIF for employment status in BE and CH (CAWI). Moreover, DIF was observed for item HLS_19_-NAV7 (find information on the quality of a particular health service) for gender and age in CZ, only for age in FR, and for education level and ‘difficulties with paying bills’ in CH (CAWI). Item HLS_19_-NAV8 (judge if a particular health service will meet your expectations and wishes on health care) displayed DIF for age in FR and for gender, age, and social status in CZ (CATI), and item HLS_19_-NAV9 (understand how to get an appointment with a particular health service) for age and employment status in AT, general health status in CH (CATI) and age in CZ (CATI). Item HLS_19_-NAV12 (stand up for yourself if your health care does not meet your needs) displayed DIF for ‘difficulties with paying bills’ in BE. For data collected in DE and SI (CAWI, CAPI) no item displayed DIF when sample size was reduced to *n* = 720.

Response dependency was observed between items HLS_19_-NAV7 (find information on the quality of a particular health service) and HLS_19_-NAV8 (judge if a particular health service will meet your expectations and wishes on health care) for data collected in BE (r = 0.37), PT (r = 0.43), and CH (r = 0.38) [47] (p. 34) (not reported in the Table). No signs of unordered response categories were observed [47].

### 3.4. Reliability

The HLS_19_-NAV shows acceptable to high internal consistency across countries (Table 7). The alpha and omega coefficient values are above 0.83 for all dichotomized data sets and above 0.88 for all polytomous data sets. The AVE is above 0.5 in all data sets except the German (AVE_dichotomous_ = 0.49, AVE_polytomous_ = 0.48) and Swiss (CATI) (AVE_polytomous_ = 0.49) data. The PSI based on dichotomized data was considerably lower than for polytomous data. However, most PSI values were still above the required target values for acceptable internal consistency (Table 7).

### 3.5. Content, Discriminant and Concurrent Predictive Validity

Content or face validity was ensured by developing the HLS_19_-NAV with regard to its underlying theoretical framework and definition of NAV-HL, as the interactional level is only reflected with item HLS_19_-NAV12 in the scale.

With respect to discriminant validity, the NAV-HL scale showed a positive moderate to high correlation with the GEN-HL scale. Correlation coefficients based on dichotomous data varied from 0.41 (BE) to 0.64 (SI, CAPI), while correlation coefficients were higher based on polytomous data with exception to CH (CATI) (Table 8).

The analysis confirms that NAV-HL is associated with sociodemographic and socioeconomic factors across countries (Table 9). Financial deprivation and self-perceived social status were significant predictors of NAV-HL in seven of the eight countries, with standardized coefficients varying between β = −0.09 (FR) and β = −0.25 (CZ) for financial deprivation and β = 0.12 (CZ) to β = 0.22 (BE) for self-perceived social status. The analyses revealed that education is negatively associated with the NAV-HL score in five countries (varying between β = −0.06 (AT) and β = 0.13 (CH)), whereas a positive association was observed in the German data (β = 0.10). NAV-HL scores decrease with increased age in some countries (ranging from β = −0.07 (AT) to β = −0.13 (FR)). For gender, no consistent pattern across countries was found. The regression models explained 4% (AT) to 13% (PT) of the variance.

Controlled for gender, age, education, self-perceived social status, and financial deprivation, NAV-HL was a significant predictor for self-reported general health status in seven of eight countries, with standardized coefficients varying between β = −0.06 (FR) and β = −0.13 (AT, DE) (Table 10). For the regression models of NAV-HL score and self-reported general health status, the explained variance varied between 12% (BE) and 32% (PT). In terms of concurrent predictive validity, it was not tested whether the use of the polytomous score affects the results. However, it can be expected from the other analyses for polytomous data that, for these, the coefficients would be somewhat higher.

## 4. Discussion

This is the first study shedding light on to what extent the newly developed HLS_19_-NAV scale has acceptable psychometric properties and validity characteristics.

Fitting a single-factor CFA (based on dichotomous and polytomous data), the HLS_19_-NAV data obtained acceptable goodness-of-fit indices across countries, confirming that it is permissible to summarize the twelve NAV-HL items in one factor score. However, Rasch modelling indicates that the HLS_19_-NAV scale is not strictly unidimensional. As the HLS_19_-NAV is based on a framework with theoretically derived levels, multidimensionality was expected to some degree. This was also confirmed by the two-factor CFA model (based on dichotomous and polytomous data), showing improved fit values in comparison to the single-factor model. Considering a reduced sample size, the HLS_19_-NAV showed sufficient overall fit to the PCM in most countries, except for FR. The NAV-HL scale had sufficient internal consistency and reliability across countries. Furthermore, targeting of the scale was sufficient in most countries, pointing to a well-balanced level of item difficulty [60,69].

Some items displayed significant misfit (applying a reduced sample size). A systematic pattern across countries was found for item HLS_19_-NAV9 (understand how to get an appointment with a particular health service) with a poor data–model fit in most countries. HLS_19_-NAV9 was also identified as an under-discriminating item in data from BE and FR, leading to the conclusion that, apparently, the item measures something else in addition to NAV-HL that is negatively correlated with the underlying concept. It might be possible that the item is not only associated with the understanding of health information, but also with difficulties in obtaining appointments, as long waiting times for health services have been an important issue across countries [70]. As item HLS_19_-NAV9 showed limitations in both Rasch analyses and in the CFA, we conclude that it may be beneficial to the scale if item HLS_19_-NAV9 is supplemented by alternative items in future studies to examine whether it can be replaced.

It was also inspected whether respondents from different sociodemographic groups but with the same location on the underlaying latent trait, NAV-HL, responded differently on the given information tasks (DIF) [71]. In most samples, some items displayed DIF for personal factors, but no consistent patterns were observed. However, since possible causes of DIF include the content of an item, its level of intricacy in words and sentences, differences in cultural relevance [72], and probably differences in HCS characteristics, a further evaluation of items showing DIF in certain population groups is recommended. This could be done, for example, by using focus groups or cognitive interviews [73]. This is also supported by the fact that, in DE, where the items were evaluated with the help of a qualitative approach in the process of instrument development, no problems with DIF were noted (with a reduced sample size).

Another objective of this study was to investigate whether and how far the use of dichotomous (as was done in HLS_19_) or polytomous (as was done in the HLS-EU) data affects the psychometric properties of the HLS_19_-NAV scale. No major differences were found between the polytomous and dichotomous responses when using CFA, with exception to higher RMSEA values when CFA was based on polytomous data. It may be considered “normal” that the presented fit indices come to different recommendations in terms of model fit as they react differently to model weaknesses, violation of distributional assumptions, or sample sizes [74] (p. 221). For this reason, Hu and Bentler [54] (pp. 27,28) (also Weiber and Mühlhaus [74] (p. 223)) recommended that, for large samples (*n* > 250), conclusions about model fit should be based on a combination of TLI or CFI (≥0.95–0.96) under consideration of the SRMR (≤0.09–0.10). Given that polytomous data also met these criteria, it is reasonable to assume that CFA describes the data sufficiently well for both polytomous and dichotomous responses. Considering that there were no problems with the four response categories of the HLS_19_-NAV and that the polytomous NAV-HL score tends to be more normally distributed than the dichotomous score across countries (Figgures S1 and S2), it may be beneficial in future studies to calculate a score based on polytomous data, since dichotomization always goes hand-in-hand with a loss of information and thus statistical power [42,75,76].

Furthermore, the analysis revealed response dependency between item HLS_19_-NAV7 (find information on the quality of a particular health service) and item HLS_19_-NAV8 (judge if a particular health service will meet your expectations and wishes on health care) in BE, CH, and PT, meaning that these items have something more in common than the underlying latent trait, which is reasonable as both items intend to measure related aspects that are important for choosing a particular health service. Although this was only a problem in three countries, it should be carefully examined whether adjustments of these “too similar” items [47] (p. 7) in the three countries will be beneficial in light of a future cross-national assessment of NAV-HL.

Regarding content or face validity, one strength of the instrument is that the HLS_19_-NAV scale was developed based on a definition and conceptual framework. Nevertheless, the interactional level of the NAV-HL framework is only represented by item HLS_19_-NAV12 (stand up for yourself if your health care does not meet your needs) in the scale [24], which implies a limitation in terms of content validity. It is likely that interacting and communicating with health services and professionals is critical for navigating the HCS [40,77]. At the same time, it must be considered that these aspects go beyond NAV-HL and form overarching concepts. For this reason, it was decided at an early development phase of the HLS_19_-NAV to display interactive tasks only very cautiously and to develop a separate instrument with a general focus on communicative HL, the HLS_19_-COM-P [42]. In future studies, it is recommended to examine whether items on interactive and communicative HL could be added to the NAV-HL scale to better reflect the conceptual NAV-HL framework. The items of the HLS_19_-COM-P may be used as a first starting point. Nevertheless, they have to be transferred to the specific field of navigating the HCS as they specifically measure general communicative HL in interaction with physicians [42].

Concerning concurrent predictive validity, the analysis revealed that HL-NAV is determined by sociodemographic and socioeconomic factors, as was shown for general HL [17,78]. NAV-HL was also linked to general health status in most countries, indicating that the measure is able to provide some kind of predictive value.

Discriminant validity as one indicator for construct validity was examined for the relationship between NAV-HL and GEN-HL. It is reasonable to conclude that, with HLS_19_-NAV, new aspects in managing health information for the specific context of navigating the HCS were assessed, since the two measures correlate only to a certain extent. At the same time, the results point to overlaps between the two measures, leading to the assumption that NAV-HL belongs to a family of specific HL measures, introduced in the HLS_19_, which are all linked to GEN-HL [17,42].

### Strengths and Limitations

The major strength of this study is that information on the psychometric properties of the newly developed HLS_19_-NAV is based on large representative population samples from eight countries and that the HLS_19_-NAV scale could be successfully applied and evaluated for different HCSs, different languages, and for different methods of data collection.

A limitation of the study is that different linguistic groups within BE and CH were merged but not considered in the analyses. Furthermore, the timing of data collection was not strictly standardized across countries. In this regard, the COVID-19 pandemic may have influenced the results. It is likely that, in most countries (except DE, where data was collected before the pandemic), respondents who used COVID-19-related services were exposed to an increased level of information about the HCS. This information may have differed in content and availability from conventional information about the HCS and may have played a role in item interpretations. Moreover, country-specific characteristics of the HCS may have an impact on how the items were interpreted. A further limitation is that responses to the HLS_19_-NAV items may not only rely on respondents’ direct experiences but also on general assessments, which is a well-known limitation of the self-assessment approach. However, self-reporting, in contrast to objective measures, offers the chance to better understand the barriers of the HCS from the user’s perspective.

## 5. Conclusions

Obstacles in navigating the HCS are burdensome for many patients and users and may increase inequities in access to and the results of health care [79,80]. For this reason, measuring NAV-HL is important for deriving recommendations for decision-makers and practitioners on where and how information and practices should be adjusted to strengthen population NAV-HL, but also for shaping user-friendly, transparent, health-literate HCSs and organisations. With the development, introduction, and validation of the new HLS_19_-NAV, a first important step towards measuring NAV-HL in general adult populations has been achieved. The new HLS_19_-NAV has proven to be a suitable instrument for measuring NAV-HL across countries, different HCSs, different languages, and different data collection methods by acceptable psychometric properties and first validation results. In addition, its integration into a family of HL measures—all introduced within HLS_19_—makes it possible to generate new specific insights about HL in a specific field without losing sight of the underlying HL concept. However, there is also room for improvement, especially with respect to under-discriminating items and DIF. In further analysis of the HLS_19_-NAV data and future studies, the instrument should be tested in specific population groups who are particularly dependent on navigation-related information, such as patients with chronic illness or caregivers.

### Instrument Use

The instrument belongs to the HLS_19_ Consortium. The use of the instrument needs contractual agreement between a non-profit applicant and the HLS_19_ Consortium. Further information can be found here: https://m-pohl.net/tools, accessed on 24 October 2022.

## Figures and Tables

**Table 1:** The HLS_19_-NAV items.

On a Scale from Very Easy to Very Difficult, How Easy Would You Say It Is to…	
HLS_19_-NAV1	…understand information on how the health care system works [e.g., which type of health services are available]
HLS_19_-NAV2	…judge which type of health service you need in case of a health problem
HLS_19_-NAV3	…judge to what extent your health insurance covers a particular health service [e.g., are there any co-payments]
HLS_19_-NAV4	…understand information on ongoing health care reforms that might affect your health care
HLS_19_-NAV5	…find out about your rights as a patient or user of the health care system
HLS_19_-NAV6	…decide for a particular health service [e.g., choose from different hospitals]
HLS_19_-NAV7	…find information on the quality of a particular health service
HLS_19_-NAV8	…judge if a particular health service will meet your expectations and wishes on health care
HLS_19_-NAV9	…understand how to get an appointment with a particular health service
HLS_19_-NAV10	…find out about support options that may help you to orientate yourself in the health care system
HLS_19_-NAV11	…locate the right contact person for your concern within a health care institution [e.g., in a hospital]
HLS_19_-NAV12	…stand up for yourself if your health care does not meet your needs

**Table 2:** Sampling procedure, mode, data collection period, and total sample size for the countries using the HLS_19_-NAV in HLS_19_.

Country	Sampling Procedure	Mode of Data Collection	Data Collection Period	Total Sample Size
AT	Multi-stage random sampling	CATI	16 March 2020–26 May 2020	2967
BE	Quota sampling	CAWI	30 January 2020–28 February 2020 and 1 October 2020–26 October 2020	1000
CH	Multi-stage random sampling	CAWI, CATI	5.March 2020–29 April 2020	2502
CZ	Random quota sampling (CAWI), Random digital procedure (CATI)	CAWI, CATI	10 November 2020–24 November 2020	1599
DE	Multi-stage random and quota sampling	PAPI	13 December 2019–27 January 2020	2143
FR	Quota sampling	CAWI	27 May 2020–5 June 2020 and 8 January 2021–18 January 2021	2003
PT	Random stratified sampling procedure	CATI	10 December 2020–13 January 2021	1247
SI	Multi-stage random sampling	CAWI, CAPI, Paper and pencil, self-administered	9 March 2020–15 March 2020 and 9 June 2020–10 August 2020	3360

AT = Austria, BE = Belgium, CH = Switzerland, CZ = Czech Republic, DE = Germany, FR = France, PT = Portugal, SI = Slovenia; CAPI = computer-assisted personal interviewing; CATI = computer-assisted telephone interviewing; CAWI = computer-assisted web interviewing; PAPI = pen-and-paper personal interview. No analysis is reported for the small, self-administered Paper-and-pencil sample (*n* = 12) in SI.

**Table 3:** Sample characteristics.

Characteristic	AT (CATI)	BE (CAWI)	CH (CAWI)	CH (CATI)	CZ (CAWI)	CZ (CATI)	DE (PAPI)	FR (CAWI)	PT (CATI)	SI (CAWI)	SI (CAPI)
*n*	2967	1000	2310	192	1067	532	2143	2003	1247	1488	1860
Characteristic mean											
Self-perceived social status (1–10)	6.3	6.5	5.9	5.6	5.7	5.6	6.0	5.7	5.4	5.7	5.2
Q25/Q75	5/7	6/7	5/7	5/7	5/7	5/7	5/7	5/7	6/7	5/7	4/6
Characteristic (in %)											
Gender											
Male	44.2	49.6	49.5	39.1	51.4	40.8	49.5	49.2	48.4	45.4	47.0
Female	55.9	50.4	50.4	60.9	48.6	59.2	50.3	50.8	51.6	54.6	53.0
Missing	/	/	0.1	/	/	/	0.2	/	/	/	/
*Age*											
18–25	6.8	9.0	10.6	0.5	12.8	1.7	9.3	12.0	13.2	11.5	5.3
26–35	12.1	20.7	15.8	1.6	22.5	12.8	13.8	17.7	14.5	19.0	9.4
36–45	15.1	14.2	17.3	4.2	21.7	4.1	15.2	19.0	20.5	21.4	13.8
46–55	23.7	22.1	21.5	8.3	17.9	8.7	16.4	19.7	18.6	18.2	16.5
56–65	19.3	17.7	16.9	15.1	14.2	24.3	19.8	18.5	17.6	17.1	22.3
66–75	13.3	13.5	11.7	35.4	9.4	35.2	13.7	13.2	10.2	8.9	19.0
76 and older	9.6	2.8	6.1	34.9	1.6	13.4	10.9	/	3.9	4.0	13.7
Missing	0.1	/	0.2	/	/		0.9	/	1.5	/	/
Level of education											
Lower secondary education or below	10.5	3.0	12.1	23.4	37.6	62.8	9.3	3.6	40.5	15.6	45.8
Higher secondary education	51.5	11.7	46.2	62.0	34.2	23.9	45.0	14.3	30.5	38.7	35.1
Above secondary education	38.0	84.1	41.3	14.6	28.2	13.2	43.6	82.1	29.0	45.7	19.1
Missing	/	1.2	0.3	/	/	0.2	2.1	/	/	/	/
Employment											
Employed or Self- Employed	56.7	53.2	65.2	24.0	65.7	32.1	56.0	61.7	61.0	62.6	42.3
Unemployed or unable to work	4.2	10.0	4.3	4.7	5.7	2.1	4.2	8.2	9.1	6.5	8.0
Other	38.8	33.0	30.1	71.4	28.6	65.8	38.7	30.0	29.5	30.9	49.7
Missing	0.2	3.8	0.4	/	/	/	1.2	0.2	0.3	0.1	0.2
Paying bills											
(Very) easy	85.7	62.4	70.8	75.0	67.4	81.4	73.6	74.6	56.7	61.2	56.2
(Very) difficult	13.4	37.6	28.6	24.0	32.6	18.2	22.5	25.4	40.7	38.7	42.5
Missing	0.9	/	0.6	1.0	/	0.4	3.8	/	2.7	0.1	1.3
Health in general											
Very good	35.3	9.0	26.0	24.0	20.9	13.4	11.9	15.4	11.4	21.3	19.4
Good	46.3	48.7	55.4	49.0	42.6	35.3	49.5	48.6	51.1	50.7	42.9
Fair (i.e., neither good nor bad)	15.4	34.4	15.6	19.8	28.0	36.8	31.7	28.6	32.2	24.3	28.0
Bad	2.46	7.0	2.5	5.7	7.8	10.9	5.9	6.7	5.1	3.2	8.1
Very bad	0.4	0.9	0.4	1.0	0.8	3.4	1.0	0.7	0.2	0.4	1.5
Missing	0.1	/	0.1	0.5	/	0.2	0.1	/	/	0.1	0.1

AT = Austria, BE = Belgium, CH = Switzerland, CZ = Czech Republic, DE = Germany, FR = France, PT = Portugal, SI = Slovenia; CAPI = computer-assisted personal interviewing; CATI = computer-assisted telephone interviewing; CAWI = computer-assisted web interviewing; PAPI = pen-and-paper personal interview. In BE and CH, different linguistic groups were merged: French, German, and Italian in CH and French and Dutch in BE. In FR, no data was collected among people aged 75+. Values are rounded to one decimal place. Table is based on unweighted data.

**Table 4:** Goodness-of-fit indices for single-factor CFA of the HLS_19_-NAV based on dichotomous and polytomous data (based on HLS_19_ Consortium [17] (p. 211)).

		AT (CATI)	BE (CAWI)	CH (CAWI)	CH (CATI)	CZ (CAWI)	CZ (CATI)	DE (PAPI)	FR (CAWI)	PT (CATI)	SI (CAWI)	SI (CAPI)
SRMR	dichotomous	0.05	0.06	0.07	0.06	0.03	0.05	0.07	0.05	0.06	0.05	0.05
	polytomous	0.04	0.06	0.07	0.07	0.03	0.04	0.07	0.05	0.06	0.05	0.05
RMSEA	dichotomous	0.05	0.07	0.07	0.00	0.02	0.00	0.06	0.06	0.07	0.05	0.05
	polytomous	0.07	0.12	0.12	0.07	0.04	0.04	0.10	0.10	0.13	0.10	0.09
RMSEA (*p* value)	dichotomous	0.48	0.00	0.00	1.00	1.00	1.00	0.00	0.01	0.00	0.13	0.30
	polytomous	0.00	0.00	0.00	0.11	0.96	0.93	0.00	0.00	0.00	0.00	0.00
CFI	dichotomous	0.99	0.99	0.99	1.00	1.00	1.00	0.98	1.00	1.00	0.99	0.99
	polytomous	0.99	0.99	0.99	0.99	1.00	1.00	0.98	0.99	0.99	0.99	0.99
TLI	dichotomous	0.99	0.99	0.98	1.00	1.00	1.00	0.97	0.99	0.99	0.99	0.99
	polytomous	0.99	0.99	0.98	0.99	1.00	1.00	0.97	0.99	0.99	0.99	0.99
GFI	dichotomous	0.99	0.99	0.99	0.99	1.00	0.99	0.98	1.00	0.99	0.99	0.99
	polytomous	1.00	0.99	0.99	0.98	1.00	1.00	0.98	1.00	0.99	0.99	1.00
AGFI	dichotomous	0.99	0.98	0.98	0.99	1.00	0.99	0.97	0.99	0.99	0.99	0.99
	polytomous	0.99	0.98	0.98	0.97	1.00	0.99	0.97	0.99	0.99	0.99	0.99

AT = Austria, BE = Belgium, CH = Switzerland, CZ = Czech Republic, DE = Germany, FR = France, PT = Portugal, SI = Slovenia; CAPI = computer-assisted personal interviewing; CATI = computer-assisted telephone interviewing; CAWI = computer-assisted web interviewing; PAPI = pen-and-paper personal interview. SRMR = standardized root-mean square residual, RMSEA = root-mean-square error of approximation, CFI = comparative fit index, TLI = Tucker–Lewis index, GFI = goodness-of-fit index, AGFI = adjusted goodness-of-fit index. CI = confidence interval.

**Table 5:** Goodness-of-fit indices for two-factor CFA of the HLS_19_-NAV based on dichotomous and polytomous data (based on HLS_19_ Consortium [17] (Annex).

		AT (CATI)	BE (CAWI)	CH (CAWI)	CH (CATI)	CZ (CAWI)	CZ (CATI)	DE (PAPI)	FR (CAWI)	PT (CATI)	SI (CAWI)	SI (CAPI)
SRMR	dichotomous	0.04	0.05	0.06	0.05	0.03	0.05	0.06	0.04	0.04	0.04	0.04
	polytomous	0.03	0.04	0.05	0.06	0.02	0.04	0.06	0.04	0.04	0.04	0.03
RMSEA	dichotomous	0.04	0.05	0.06	0.00	0.00	0.00	0.06	0.05	0.04	0.04	0.03
	polytomous	0.05	0.10	0.11	0.06	0.03	0.04	0.09	0.09	0.10	0.08	0.06
RMSEA (*p* value)	dichotomous	0.94	0.39	0.00	1.00	1.00	1.00	0.01	0.32	0.88	0.98	1.00
	polytomous	0.38	0.00	0.00	0.35	1.00	0.81	0.00	0.00	0.00	0.00	0.00
CFI	dichotomous	0.99	1.00	0.99	1.00	1.00	1.00	0.98	1.00	1.00	1.00	1.00
	polytomous	1.00	0.99	0.99	0.99	1.00	1.00	0.98	1.00	1.00	1.00	1.00
TLI	dichotomous	0.99	0.99	0.99	1.00	1.00	1.00	0.98	1.00	1.00	1.00	1.00
	polytomous	1.00	0.99	0.99	0.99	1.00	1.00	0.98	1.00	1.00	1.00	1.00
GFI	dichotomous	0.99	0.99	0.99	0.99	1.00	0.99	0.98	1.00	1.00	1.00	1.00
	polytomous	1.00	0.99	0.99	0.99	1.00	1.00	0.99	1.00	1.00	1.00	1.00
AGFI	dichotomous	0.99	0.99	0.98	0.99	1.00	0.99	0.98	0.99	1.00	1.00	1.00
	polytomous	1.00	0.99	0.98	0.98	1.00	0.99	0.98	0.99	0.99	0.99	1.00

AT = Austria, BE = Belgium, CH = Switzerland, CZ = Czech Republic, DE = Germany, FR = France, PT = Portugal, SI = Slovenia; CAPI = computer-assisted personal interviewing; CATI = computer-assisted telephone interviewing; CAWI = computer-assisted web interviewing; PAPI = pen-and-paper personal interview. SRMR = standardized root-mean square residual, RMSEA = root-mean-square error of approximation, CFI = comparative fit index, TLI = Tucker–Lewis index, GFI = goodness-of-fit index, AGFI = adjusted goodness-of-fit index. CI = confidence interval.

**Table 6:** Overall data–model fit for the polytomous HLS_19_-NAV data when fitted against the partial credit parametrization of the unidimensional Rasch model (taken from Guttersrud et al. [47] (p. 15)).

	AT (CATI)	BE (CAWI)	CH (CAWI)	CH (CATI)	CZ (CAWI)	CZ (CATI)	DE (PAPI)	FR (CAWI)	PT (CATI)	SI (CAWI)	SI (CAPI)
χ^2^ *(p)*	59.3 (0.130)	107.3 (<0.001 **)	74.3 (0.010 *)	75.37 (0.010 *)	73.4 (0.010 *)	80.5 (<0.00 **)	73.8 (0.010 *)	165.2 (<0.001 **)	122.9 (<0.001 **)	137.5 (<0.001 **)	105.0 (<0.001 **)
Mean person location	0.91	−0.07	0.04	0.54	−0.15	0.52	−0.31	0.11	0.21	0.96	0.63
Dimensionality, % sign. tests	9.6	11.5	9.7	7.4	5.3	4.2	12.2	8.3	9.3	9.2	10.3

AT = Austria, BE = Belgium, CH = Switzerland, CZ = Czech Republic, DE = Germany, FR = France, PT = Portugal, SI = Slovenia; CAPI = computer-assisted personal interviewing; CATI = computer-assisted telephone interviewing; CAWI = computer-assisted web interviewing; PAPI = pen-and-paper personal interview. * *p* < 0.05, ** *p* < 0.01. The chi-square (χ^2^) test for overall data–model fit was based on reduced sample sizes with 20 persons per threshold (*n* = 720).

**Table 7:** Cronbach’s alpha, Person Separation Index (PSI), omega, and average variance extracted (AVE) for the HLS_19_-NAV based on dichotomous and polytomous data (partly taken from Guttersrud et al. [47] (p. 15) and HLS_19_ Consortium [17] (p. 213)).

		AT (CATI)	BE (CAWI)	CH (CAWI)	CH (CATI)	CZ (CAWI)	CZ (CATI)	DE (PAPI)	FR (CAWI)	PT (CATI)	SI (CAWI)	SI (CAPI)
Alpha	dichotomous	0.88	0.90	0.88	0.89	0.90	0.87	0.83	0.91	0.92	0.90	0.90
	polytomous	0.92	0.93	0.92	0.88	0.93	0.91	0.88	0.94	0.94	0.94	0.93
PSI	dichotomous	0.68	0.80	0.78	0.68	0.77	0.70	0.73	0.81	0.73	0.74	0.73
	polytomous	0.90	0.92	0.91	0.83	0.92	0.88	0.88	0.93	0.88	0.92	0.91
Omega	dichotomous	0.88	0.91	0.89	0.90	0.91	0.88	0.84	0.92	0.93	0.91	0.91
	polytomous	0.92	0.94	0.92	0.88	0.93	0.91	0.89	0.94	0.94	0.94	0.93
AVE	dichotomous	0.59	0.66	0.61	0.63	0.65	0.57	0.49	0.71	0.76	0.69	0.68
	polytomous	0.58	0.63	0.61	0.49	0.63	0.55	0.48	0.67	0.72	0.66	0.65

AT = Austria, BE = Belgium, CH = Switzerland, CZ = Czech Republic, DE = Germany, FR = France, PT = Portugal, SI = Slovenia; CAPI = computer-assisted personal interviewing; CATI = computer-assisted telephone interviewing; CAWI = computer-assisted web interviewing; PAPI = pen-and-paper personal interview.

**Table 8:** Pearson correlation between the HLS_19_-NAV scores and the HLS_19_-Q12 scores based on dichotomous and polytomous data (based on HLS_19_ Consortium [17] (p. 214)).

		AT (CATI)	BE (CAWI)	CH (CAWI)	CH (CATI)	CZ (CAWI)	CZ (CATI)	DE (PAPI)	FR (CAWI)	PT (CATI)	SI (CAWI)	SI (CAPI)
NAV-HL and GEN-HL	dichotomous	0.56	0.41	0.56	0.52	0.53	0.57	0.60	0.63	0.53	0.60	0.64
	polytomous	0.59	0.42	0.63	0.49	0.56	0.61	0.64	0.70	0.58	0.65	0.69

AT = Austria, BE = Belgium, CH = Switzerland, CZ = Czech Republic, DE = Germany, FR = France, PT = Portugal, SI = Slovenia; CAPI = computer-assisted personal interviewing; CATI = computer-assisted telephone interviewing; CAWI = computer-assisted web interviewing; PAPI = pen-and-paper personal interview.

**Table 9:** Multivariable linear regression models of NAV-HL score (dependent variable) by social determinants (independent variables) for total samples in countries (equally weighted) (mainly taken from HLS_19_ Consortium [17] (p. 217)).

	AT		BE		CH		CZ		DE		FR		PT		SI	
	b	β	b	β	b	β	b	β	b	β	b	β	b	β	b	β
Intercept	88.28		28.56		58,98		58.79		31.32		65.81		66.81		77.14	
Gender female	−1.00	−0.02	−3.38	−0.05	−2.60	−0.04	1.59	0.02	−0.32	−0.01	−4.44	**−0.07**	−0.26	−0.00	0.51	0.01
Age in years	−0.14	**−0.07**	−0.05	−0.02	0.02	0.01	0.04	−0.02	−0.14	**−0.09**	−0.29	**−0.13**	−0.20	**−0.10**	−0.17	**−0.09**
Education	−1.02	**−0.06**	−0.51	−0.03	−2.22	**−0.13**	−2.58	**−0.14**	1.69	**0.10**	−2.24	**−0.10**	−1.27	**−0.08**	−0.45	−0.02
Self-perceived social status	0.09	0.00	4.71	**0.22**	2.58	**0.14**	2.59	**0.12**	2.80	**0.15**	3.74	**0.17**	4.19	**0.18**	2.38	**0.12**
Financial deprivation	−7.01	**−0.18**	−0.27	−0.01	−4.72	**−0.17**	−7.89	**−0.25**	−3.10	**−0.11**	−3.08	**−0.09**	−6.19	**−0.23**	−5.88	**−0.23**
R^2^	0.04		0.05		0.07		0.1		0.09		0.06		0.13		0.11	
Valid count	2587		988		1983		1523		1845		2003		1012		3160	
Total count	2967		1000		2502		1599		2143		2003		1247		3360	

Standardized coefficients (β) with *p*-values lower than 0.01 in bold. Education by 9 ISCED levels, from 0 (lowest) to 8 (highest level). Self-perceived social status (from 1 = lowest level to 10 = highest level in society). Financial deprivation: 4 categories, from no deprivation (0) to severe deprivation (100). Due to rounding the numbers to two significant decimals, ±0.00 may represent a value <0.005.

**Table 10:** Multivariable linear regression models of self-reported general health status (dependent variable) by NAV-HL score and other social variables (independent variables) (equally weighted) (mainly taken from HLS_19_ Consortium [17] (p. 223)).

	AT		BE		CH		CZ		DE		FR		PT		SI	
	b	β	b	β	b	β	b	β	b	β	b	β	b	β	b	β
Intercept	1.81		3.41		2.10		1.96		1.78		2.29		1.71		1.50	
NAV-HL	−0.00	**−0.13**	−0.00	**−0.10**	−0.00	**−0.1**	−0.00	**−0.07**	−0.00	**−0.13**	−0.00	**−0.06**	−0.00	−0.01	−0.00	**−0.12**
gender	−0.03	−0.02	0.06	0.04	−0.07	−0.05	−0.05	−0.03	−0.05	−0.03	−0.03	−0.02	0.13	**0.09**	0.04	0.02
Age in years	0.01	**0.24**	0.00	0.07	0.01	**0.22**	0.02	**0.35**	0.02	**0.41**	0.01	**0.23**	0.01	**0.33**	0.02	**0.37**
Education	−0.03	**−0.06**	−0.03	−0.08	−0.02	−0.04	−0.06	**−0.11**	−0.01	−0.03	0.01	0.02	−0.05	**−0.13**	−0.03	**−0.08**
Self-perceived social status	−0.06	**−0.11**	−0.14	**−0.28**	−0.08	**−0.18**	−0.08	**−0.13**	−0.04	**−0.08**	−0.12	**−0.23**	−0.06	**−0.11**	−0.04	**−0.07**
Financial deprivation	0.16	**0.16**	−0.02	−0.04	0.10	**0.16**	0.13	**0.15**	0.11	**0.13**	0.10	**0.12**	0.11	**0.18**	0.13	**0.19**
R^2^	0.15		0.12		0.15		0.25		0.26		0.15		0.32		0.3	
Valid count	2584		988		1982		1523		1843		2003		1012		3157	
Total count	2967		1000		2502		1599		2143		2003		1247		3360	

Standardized coefficients (β) with *p*-values lower than 0.01 in bold. NAV-HL score, from 0 (minimal) to 100 (maximal). Education by 9 ISCED levels, from 0 (lowest) to 8 (highest level). Self-perceived social status (from 1 = lowest level to 10 = highest level in society). Financial deprivation: 4 categories, from no deprivation (0) to severe deprivation (100). Due to rounding the numbers to two significant decimals, ±0.00 may represent a value <0.005.

## Data Availability

Information about data supporting reported results can be found on the M-POHL webpage, https://m-pohl.net/Design_Methods, accessed on 24 October 2022.

## Supplementary Materials

The following supporting information can be downloaded at: https://www.mdpi.com/article/10.3390/ijerph192113863/s1, Table S1: Categories used for the analysis of differential item functioning (DIF); Table S2: Entries in the residual correlation matrix >0.10 based on dichotomous data; Table S3: Entries in the residual correlation matrix >0.10 based on polytomous data; Table S4: Power of fit and extreme records in Rasch modelling based on dichotomous and polytomous (italic) data; Table S5–S15: Item fit statistics of the HLS_19_-NAV for Austria (CATI), Belgium (CAWI), Czech Republic (CAWI, CATI), France (CAWI), Germany (PAPI), Portugal (CATI), Slovenia (CAWI, CAPI), and Switzerland (CAWI, CATI); Figure S1: Distribution of the NAV-HL score (0–100) based on dichotomous data; Figure S2: Distribution of the NAV-HL score (0–100) based on polytomous data.

## References

[1] van der Aa M.J., van den Broeke J.R., Stronks K., Plochg T. (2017). Patients with multimorbidity and their experiences with the healthcare process: A scoping review. J. Comorb..

[2] Gui X., Chen Y., Pine K.H. (2018). Navigating the Healthcare Service “Black Box”. Proc. ACM Hum. Comput. Interact..

[3] Lee E.S., Muthulingam G., Chew E.A.L., Lee P.S.S., Koh H.L., Quak S.X.E., Ding Y.Y., Subramaniam M., Vaingankar J.A. (2020). Experiences of older primary care patients with multimorbidity and their caregivers in navigating the healthcare system: A qualitative study protocol. J. Comorb..

[4] Rein A. Navigating Health Care: Why It’s So Hard and What Can Be Done to Make It Easier for the Average Consumer. http://www.hcfo.org/files/hcfo/HCFONavigatingHealthCare.pdf.

[5] Schaeffer D., Haslbeck J., Hurrelmann K., Richter M. (2016). Bewältigung chronischer Krankheit. Soziologie von Gesundheit und Krankheit.

[6] Schnitzer S., Kohl R., Fügemann H., Gödde K., Stumm J., Engelmann F., Grittner U., Rieckmann N. (2022). Patient Navigation-Who Needs What? Awareness of Patient Navigators and Ranking of Their Tasks in the General Population in Germany. Int. J. Environ. Res. Public Health.

[7] Sofaer S. (2009). Navigating poorly charted territory: Patient dilemmas in health care “nonsystems”. Med. Care Res. Rev..

[8] Bodenheimer T. (2008). Coordinating care-a perilous journey through the health care system. N. Engl. J. Med..

[9] Hofmarcher M.M., Oxley H., Rusticelli E. (2007). Improved Health System Performance through Better Care Coordination: OECD Health Working Papers.

[10] McKenney K.M., Martinez N.G., Yee L.M. (2018). Patient navigation across the spectrum of women’s health care in the United States. Am. J. Obstet. Gynecol..

[11] Schaeffer D., Hurrelmann K., Bauer U., Kolpatzik K. (2018). National Action Plan Health Literacy. Promoting Health Literacy in Germany.

[12] Snelgrove S., Liossi C. (2013). Living with chronic low back pain: A metasynthesis of qualitative research. Chronic Illn..

[13] Schwarz T., Schmidt A.E., Bobek J., Ladurner J. (2022). Barriers to accessing health care for people with chronic conditions: A qualitative interview study. BMC Health Serv. Res..

[14] (2018). Sachverständigenrat zur Begutachtung der Entwicklung im Gesundheitswesen. Bedarfsgerechte Steuerung der Gesundheitsversorgung.

[15] Schaeffer D., Pelikan J.M., Schaeffer D., Pelikan J.M. (2017). Health Literacy: Begriff, Konzept, Relevanz. Health Literacy: Forschungsstand und Perspektiven.

[16] Schaeffer D., Schaeffer D., Pelikan J.M. (2017). Chronische Krankheit und Health Literacy. Health Literacy: Forschungsstand und Perspektiven.

[17] The HLS19 Consortium of the WHO Action Network M-POHL (2021). International Report on the Methodology, Results, and Recommendations of the European Health Literacy Population Survey 2019–2021 (HLS19) of M-POHL.

[18] HLS-EU Consortium (2012). Comparative Report of Health Literacy in Eight EU Member States. The European Health Literacy Survey HLS-EU.

[19] Schaeffer D., Berens E.-M., Vogt D., Gille S., Griese L., Klinger J., Hurrelmann K. (2021). Health literacy in Germany-findings of a representative follow-up survey. Dtsch. Arztebl. Int..

[20] Speros C.I. (2009). More than words: Promoting health literacy in older adults. Online J. Issues Nurs..

[21] Bargfrede A. (2011). Patienten auf der Suche: Orientierungsarbeit im Gesundheitswesen.

[22] Robinson K.M., Christensen K.B., Ottesen B., Krasnik A. (2012). Diagnostic delay, quality of life and patient satisfaction among women diagnosed with endometrial or ovarian cancer: A nationwide Danish study. Qual. Life Res..

[23] Storla D.G., Yimer S., Bjune G.A. (2008). A systematic review of delay in the diagnosis and treatment of tuberculosis. BMC Public Health.

[24] Griese L., Berens E.-M., Nowak P., Pelikan J.M., Schaeffer D. (2020). Challenges in Navigating the Health Care System: Development of an Instrument Measuring Navigation Health Literacy. Int. J. Environ. Res. Public Health.

[25] Corbin J., Strauss A. (1988). Unending Work and Care: Managing Chronic Illness at Home.

[26] Corbin J., Strauss A. (2010). Weiterleben Lernen: Verlauf und Bewältigung Chronischer Krankheit.

[27] Nolte E., McKee M., Nolte E., McKee M. (2008). Caring for people with chronic conditions: An introduction. Caring for People with Chronic Conditions. A Health System Perspective.

[28] Schaeffer D. (2004). Der Patient als Nutzer: Krankheitsbewältigung und Versorgungsnutzung im Verlauf Chronischer Krankheit.

[29] Schaeffer D., Schaeffer D. (2009). Bewältigung chronischer Krankheit im Lebenslauf—Einleitung. Bewältigung Chronischer Krankheit im Lebenslauf.

[30] Griese L., Schaeffer D., Berens E.-M. (2022). Navigational health literacy among people with chronic illness. Chronic Illn..

[31] Ellen M.E., Wilson M.G., Vélez M., Shach R., Lavis J.N., Grimshaw J.M., Moat K.A. (2018). Addressing overuse of health services in health systems: A critical interpretive synthesis. Health Res. Policy Syst..

[32] Rasu R.S., Bawa W.A., Suminski R., Snella K., Warady B. (2015). Health Literacy Impact on National Healthcare Utilization and Expenditure. Int. J. Health Policy Manag..

[33] Parker R., Ratzan S.C. (2010). Health literacy: A second decade of distinction for Americans. J. Health Commun..

[34] Kakar R., Combs R.M., Young M.H., Ali N., Muvuka B. (2022). Health Insurance Literacy Perceptions and the Needs of a Working-Class Community. HLRP Health Lit. Res. Pract..

[35] Schmidt-Kaehler S., Schaeffer D., Pelikan J. (2019). Transfer zu einem nutzerfreundlichen und gesundheitskompetenten Gesundheitssystem. Monit. Versorg..

[36] Rudd R.E. (2004). Navigating Hospitals. Lit. Harvest.

[37] Rudd R., Kirsch I., Yamamoto K. (2004). Literacy and Health in America.

[38] Rudd E.R., Schaeffer D., Pelikan J.M. (2017). Health Literacy Developments, Corrections, and Emerging Themes. Health Literacy: Forschungsstand und Perspektiven.

[39] van der Heide I., Uiters E., Sørensen K., Röthlin F., Pelikan J., Rademakers J., Boshuizen H. (2016). Health literacy in Europe: The development and validation of health literacy prediction models. Eur. J. Public Health.

[40] Osborne R., Batterham R.W., Elsworth G.R., Hawkins M., Buchbinder R. (2013). The grounded psychometric development and initial validation of the Health Literacy Questionnaire (HLQ). BMC Public Health.

[41] Sørensen K., van den Broucke S., Fullam J., Doyle G., Pelikan J., Slonska Z., Brand H. (2012). Health literacy and public health: A systematic review and integration of definitions and models. BMC Public Health.

[42] Finbråten H.S., Nowak P., Griebler R., Bíró É., Vrdelja M., Charafeddine R., Griese L., Bøggild H., Schaeffer D., Link T. (2022). The HLS19-COM-P, a New Instrument for Measuring Communicative Health Literacy in Interaction with Physicians: Development and Validation in Nine European Countries. Int. J. Environ. Res. Public Health.

[43] Schaeffer D., Berens E.-M., Gille S., Griese L., Klinger J., de Sombre S., Vogt D., Hurrelmann K. (2021). Gesundheitskompetenz der Bevölkerung in Deutschland vor und Während der Corona Pandemie: Ergebnisse des HLS-GER 2.

[44] Adler N.E., Epel E.S., Castellazzo G., Ickovics J.R. (2000). Relationship of subjective and objective social status with psychological and physiological functioning: Preliminary data in healthy, white women. Health Psychol..

[45] Schneider S.L. (2016). The Conceptualisation, Measurement, and Coding of Education in German and Cross-National Surveys. GESIS Survey Guidelines.

[46] UNECSCO (2012). International Standard Classification of Education.

[47] Guttersrud Ø., Le C., Pettersen K.S., Finbråten H.S. Rasch Analyses of Data Collected in 17 Countries—A Technical Report to Support Decision-Making within the M-POHL Consortium: International Population Health Literacy Survey 2019–2021 (HLS19). https://m-pohl.net/sites/m-pohl.net/files/inline-files/Guttersrud%20et%20al_Rasch%20analyses%20of%20data%20colllected%20in%2017%20countries_2021_0.pdf.

[48] Sørensen K., van den Broucke S., Pelikan J., Fullam J., Doyle G., Slonska Z., Kondilis B., Stoffels V., Osborne R.H., Brand H. (2013). Measuring health literacy in populations: Illuminating the design and development process of the European Health Literacy Survey Questionnaire (HLS-EU-Q). BMC Public Health.

[49] Rosseel Y. (2012). lavaan: An R Package for Structural Equation Modeling. J. Stat. Softw..

[50] Beaujean A.A. (2014). Latent Variable Modeling Using R.

[51] Kline R.B. (2015). Principles and Practice of Structural Equation Modeling.

[52] Rosseel Y. The Lavaan Tutorial. https://lavaan.ugent.be/tutorial/tutorial.pdf.

[53] Prudon P. (2015). Confirmatory factor analysis: A brief introduction and critique. Compr. Psychol..

[54] Hu L., Bentler P.M. (1999). Cutoff criteria for fit indexes in covariance structure analysis: Conventional criteria versus new alternatives. Struct. Equ. Model. Multidiscip. J..

[55] Andrich D., Sheridan B. (2019). RUMM2030 Plus.

[56] Adams R.J., Wu M.L., Cloney D., Wilson M.R. (2020). ACER ConQuest.

[57] Masters G.N., van der Linden W.J. (2016). Partial credit model. Handbook of Item Response Theory.

[58] Smith E.V. (2002). Detecting and evaluating the impact of multidimensionality using item fit statistics and principal component analysis of residuals. J. Appl. Meas..

[59] Lamoureux E.L., Pesudovs K., Pallant J.F., Rees G., Hassell J.B., Caudle L.E., Keeffe J.E. (2008). An evaluation of the 10-item vision core measure 1 (VCM1) scale (the Core Module of the Vision-Related Quality of Life scale) using Rasch analysis. Ophthalmic Epidemiol..

[60] Tennant A., Conaghan P.G. (2007). The Rasch measurement model in rheumatology: What is it and why use it? When should it be applied, and what should one look for in a Rasch paper?. Arthritis Rheum..

[61] Wright B.D., Linacre J.M., Gustafson J.E., Martin-Lof P. (1994). Reasonable mean-square fit values. Rasch Meas. Trans..

[62] Andrich D., Marais I. (2019). A Course in Rasch Measurement Theory.

[63] Bentler P.M., Bonett D.G. (1980). Significance tests and goodness of fit in the analysis of covariance structures. Psychol. Bull..

[64] Marais I., Christensen K.B., Kreiner S., Mesbah M. (2012). Local Dependence. Rasch Models in Health.

[65] Ahmad S., Zulkurnain N., Khairushalimi F. (2016). Assessing the Validity and Reliability of a Measurement Model in Structural Equation Modeling (SEM). J. Adv. Math. Comput. Sci..

[66] Hubley A.M., Michalos A.C. (2014). Discriminant Validity. Encyclopedia of Quality of Life and Well-Being Research.

[67] Browne M.W., Cudeck R., Bollen K.A., Long J.S. (1993). Alternative ways of assessing model fit. Testing Structural Equation Models.

[68] Knekta E., Runyon C., Eddy S. (2019). One Size Doesn’t Fit All: Using Factor Analysis to Gather Validity Evidence When Using Surveys in Your Research. CBE Life Sci. Educ..

[69] Finger R.P., Fenwick E., Pesudovs K., Marella M., Lamoureux E.L., Holz F.G. (2012). Rasch analysis reveals problems with multiplicative scoring in the macular disease quality of life questionnaire. Ophthalmology.

[70] Organisation for economic cooperation and development (OECD) (2020). Waiting Times for Health Services.

[71] Holland P.W., Wainer H. (1993). Differential Item Functioning.

[72] Allalouf A., Hambleton R.K., Sireci S.G. (1999). Identifying the causes of translation DIF on verbal items. J. Educ. Meas..

[73] Teresi J.A., Fleishman J.A. (2007). Differential item functioning and health assessment. Qual. Life Res..

[74] Weiber R., Mühlhaus D. (2014). Strukturgleichungsmodellierung.

[75] Fedorov V., Mannino F., Zhang R. (2009). Consequences of dichotomization. Pharm. Stat..

[76] Irwin J.R., McClelland G.H. (2003). Negative Consequences of Dichotomizing Continuous Predictor Variables. J. Mark. Res..

[77] Carter N., Valaitis R.K., Lam A., Feather J., Nicholl J., Cleghorn L. (2018). Navigation delivery models and roles of navigators in primary care: A scoping literature review. BMC Health Serv. Res..

[78] Rowlands G., Shaw A., Jaswal S., Smith S., Harpham T. (2015). Health literacy and the social determinants of health: A qualitative model from adult learners. Health Promot. Int..

[79] Marmot M. (2020). Health equity in England: The Marmot review 10 years on. BMJ.

[80] World Health Organization (WHO)—Regional Office for Europe (2013). Health Literacy. The Solid Facts.

