# Supplementary material for: HLS19-NAV—Validation of a New Instrument Measuring Navigational Health Literacy in Eight European Countries
Source: Int J Environ Res Public Health. 2022 Oct 25;19(21):13863. doi: 10.3390/ijerph192113863 (PMC9654211; doi:10.3390/ijerph192113863)
Supplement: Supplementary file 1 [file ijerph-19-13863-s001.zip › ijerph-1971651-supplementary.pdf]

International Journal of
Environmental Research and Public Health

MDPI

Supplementary File

Tables S1 and S5–S14 (except Table S8) are taken from Guttersrud et al. [47].

Table S1. Categories used for the analysis of differential item functioning (DIF).

	Categories		
	1	2	3
Gender	Male	female	
Age [a]			
agedico	18 to 45 years	46 years or older	
agecat1	18 to 25 years	26 to 65 years	66 years or older
agecat2	18 to 45 years	46 to 75 years	76 years or older
Education	ISCED 0–3	ISCED 4–8	
Employment	employed	unemployed or retired	
Paying bills	easy	difficult	
Social level	levels 1–4	levels 5–10	
General health	(very) good or fair	(very) bad	

[a] Different age categories were used to explore if the categorization had an impact on the results in these analyses.

Table S2. Entries in the residual correlation matrix >0.10 based on dichotomous data.

Item pair	AT (CATI)	BE (CAWI)	CH (CAWI)	CH (CATI)	CZ (CAWI)	CZ (CATI)	DE (PAPI)	FR (CAWI)	PT (CATI)	SI (CAWI)	SI (CAPI)
HLS$_{19}$-NAV2 and HLS$_{19}$-NAV1	0.13	0.16	0.19			-0.11	0.16	0.17	0.11	0.15	
HLS$_{19}$-NAV7 and HLS$_{19}$-NAV1			-0.13	-0.15					-0.13	-0.11	-0.11
HLS$_{19}$-NAV8 and HLS$_{19}$-NAV1		-0.11	-0.16				-0.12			-0.11	
HLS$_{19}$-NAV7 and HLS$_{19}$-NAV2		-0.12	-0.13				-0.12	-0.13			
HLS$_{19}$-NAV8 and HLS$_{19}$-NAV7	0.14		0.16				0.19				
HLS$_{19}$-NAV9 and HLS$_{19}$-NAV2		0.11		0.12				0.13			
HLS$_{19}$-NAV9 and HLS$_{19}$-NAV11							0.11				-0.11
HLS$_{19}$-NAV4 and HLS$_{19}$-NAV3			0.13				0.11				
HLS$_{19}$-NAV7 and HLS$_{19}$-NAV5		-0.15		-0.13					-0.12		
HLS$_{19}$-NAV5 and HLS$_{19}$-NAV3		0.11	0.11			0.11					
HLS$_{19}$-NAV8 and HLS$_{19}$-NAV3			-0.14								
HLS$_{19}$-NAV8 and HLS$_{19}$-NAV4			-0.11				-0.12				
HLS$_{19}$-NAV7 and HLS$_{19}$-NAV3	-0.12										
HLS$_{19}$-NAV9 and HLS$_{19}$-NAV7		-0.12									

HLS$_{19}$-NAV9 and HLS$_{19}$-NAV8		-0.11									
HLS$_{19}$-NAV8 and HLS$_{19}$-NAV2			-0.13								
HLS$_{19}$-NAV7 and HLS$_{19}$-NAV4			-0.12								
HLS$_{19}$-NAV9 and HLS$_{19}$-NAV4			-0.12			-0.19					
HLS$_{19}$-NAV7 and HLS$_{19}$-NAV6							0.17				0.11
HLS$_{19}$-NAV6 and HLS$_{19}$-NAV4				-0.14			-0.14				
HLS$_{19}$-NAV8 and HLS$_{19}$-NAV6							0.14				
HLS$_{19}$-NAV6 and HLS$_{19}$-NAV1							-0.12				
HLS$_{19}$-NAV9 and HLS$_{19}$-NAV12					-0.14			-0.13			
HLS$_{19}$-NAV5 and HLS$_{19}$-NAV10									-0.12		
HLS$_{19}$-NAV9 and HLS$_{19}$-NAV10									0.11		
HLS$_{19}$-NAV3 and HLS$_{19}$-NAV10									-0.11		
HLS$_{19}$-NAV2 and HLS$_{19}$-NAV11				-0.12					-0.11		
HLS$_{19}$-NAV12 and HLS$_{19}$-NAV1										-0.11	
HLS$_{19}$-NAV4 and HLS$_{19}$-NAV11						0.12					-0.12
HLS$_{19}$-NAV3 and HLS$_{19}$-NAV11				-0.14		-0.17					
HLS$_{19}$-NAV9 and HLS$_{19}$-NAV1				-0.12							
HLS19-NAV12 and HLS19-NAV11											0.11

Table only contains item pairs with entries >0.10.

Table S3. Entries in the residual correlation matrix >0.10 based on polytomous data.

Item pair	AT (CATI)	BE (CAWI)	CH (CAWI)	CH (CATI)	CZ (CAWI)	CZ (CATI)	DE (PAPI)	FR (CAWI)	PT (CATI)	SI (CAWI)	SI (CAPI)
HLS$_{19}$-NAV2 and HLS$_{19}$-NAV1		0.16	0.18				0.16	0.17	0.14	0.14	
HLS$_{19}$-NAV7 and HLS$_{19}$-NAV1			-0.12	-0.17			-0.11		-0.11		
HLS$_{19}$-NAV8 and HLS$_{19}$-NAV1		-0.12	-0.14				-0.12				
HLS$_{19}$-NAV7 and HLS$_{19}$-NAV2		-0.13	-0.14						-0.11		
HLS$_{19}$-NAV8 and HLS$_{19}$-NAV7	0.12	0.1	0.15	0.16			0.16				
HLS$_{19}$-NAV9 and HLS$_{19}$-NAV2		0.14						0.12			

HLS$_{19}$-NAV9 and HLS$_{19}$-NAV11			-0.13			0.11				
HLS$_{19}$-NAV4 and HLS$_{19}$-NAV3		0.11				0.13				
HLS$_{19}$-NAV7 and HLS$_{19}$-NAV5	-0.11		-0.11					-0.11		
HLS$_{19}$-NAV8 and HLS$_{19}$-NAV3		-0.12				-0.11				
HLS$_{19}$-NAV8 and HLS$_{19}$-NAV4		-0.11				-0.12				
HLS$_{19}$-NAV7 and HLS$_{19}$-NAV3		-0.11				-0.12				
HLS$_{19}$-NAV9 and HLS$_{19}$-NAV7	-0.12									
HLS$_{19}$-NAV9 and HLS$_{19}$-NAV8	-0.12							-0.11		
HLS$_{19}$-NAV8 and HLS$_{19}$-NAV2	-0.12	-0.12								
HLS$_{19}$-NAV7 and HLS$_{19}$-NAV4		-0.11								
HLS$_{19}$-NAV9 and HLS$_{19}$-NAV4	-0.12				-0.11					
HLS$_{19}$-NAV7 and HLS$_{19}$-NAV6						0.12				
HLS$_{19}$-NAV6 and HLS$_{19}$-NAV4						0.12				
HLS$_{19}$-NAV8 and HLS$_{19}$-NAV6						0.11				
HLS$_{19}$-NAV6 and HLS$_{19}$-NAV1						-0.11				
HLS$_{19}$-NAV9 and HLS$_{19}$-NAV12		0.13	0.16				-0.12			
HLS$_{19}$-NAV9 and HLS$_{19}$-NAV10			-0.12							
HLS$_{19}$-NAV3 and HLS$_{19}$-NAV10								-0.11		
HLS$_{19}$-NAV2 and HLS$_{19}$-NAV11								-0.13		
HLS$_{19}$-NAV4 and HLS$_{19}$-NAV11										-0.12
HLS$_{19}$-NAV2 and HLS$_{19}$-NAV10			0.11							
HLS$_{19}$-NAV11 and HLS$_{19}$-NAV10			0.2							
HLS$_{19}$-NAV9 and HLS$_{19}$-NAV3			-0.17							
HLS$_{19}$-NAV6 and HLS$_{19}$-NAV10			-0.16							
HLS$_{19}$-NAV4 and HLS$_{19}$-NAV12			0.15							-0.11
HLS$_{19}$-NAV2 and HLS$_{19}$-NAV12			-0.11				-0.11			

Table only contains item pairs with entries >0.10.

Table S4. Power of fit and number of persons with extreme scores (Rasch modelling) based on dichotomous and polytomous (italic) data.

		AT (CATI)	BE (CAWI)	CH (CAWI)	CZ (CAWI)	DE (PAPI)	FR (CAWI)	PT (CATI)	SI (CAWI)	SI (CAPI)
Power of fit	dichotomous	Reasonable	Good	Good	Good	Reasonable	Good	Reasonable	Good	Good
	polytomous	*excellent*	*excellent*	*excellent*	*excellent*	*excellent*	*excellent*	*excellent*	*excellent*	*excellent*
Extreme records (n)	dicho-tomous	805	224	453	302	301	518	405	594	503
	polytomous	*101*	*23*	*56*	*41*	*29*	*78*	*21*	*62*	*57*

Table S5. Item fit statistics of the HLS_{19}-NAV for Austria (CATI).

Item	Fit.res.	Chi sq n=1080	Chi sq p	Infit MNSQ	DIF n=1080
HLS_{19}-NAV1	1.438	0.699	0.951	1.04	
HLS_{19}-NAV2	-3.111	4.67	0.323	0.95	
HLS_{19}-NAV3	3.355	7.469	0.113	1.11	agecat1 agecat2 employment
HLS_{19} -NAV4	-2.634	2.824	0.588	0.96	
HLS_{19} -NAV5	-5.544	13.339	0.010	0.89	
HLS_{19}-NAV6	-2.413	1.477	0.831	0.97	
HLS_{19}-NAV7	-2.119	2.221	0.695	0.96	education
HLS_{19}-NAV8	-2.535	3.229	0.520	0.96	
HLS_{19}-NAV9	6.008	36.788	<0.001*	1.24	agedico, agecat1* agecat2* employment*
HLS_{19}-NAV10	-5.158	10.881	0.028	0.88	
HLS_{19}-NAV11	-1.749	0.883	0.927	0.98	
HLS_{19}-NAV12	1.831	4.538	0.338	1.09	

*significant when sample size n = 720

Table S6 Item fit statistics of the HLS_{19}-NAV for Belgium (CAWI)

Item	Fit.res.	Chi sq<br>n = 1080	Chi sq p	Infit MNSQ	DIF<br>n = 1080
HLS_{19}-NAV1	-0.814	7.042	0.134	0.99	
HLS_{19}-NAV2	-0.585	8.495	0.075	1.01	
HLS_{19}-NAV3	2.195	10.005	0.040	1.15	agedico, agecat1, agecat2, employment*
HLS_{19} -NAV4	-3.166	11.127	0.025	0.91	
HLS_{19} -NAV5	-4.285	13.475	0.009	0.87	
HLS_{19}-NAV6	-0.973	2.585	0.630	1.03	
HLS_{19}-NAV7	-2.007	4.300	0.367	0.96	agedico, agecat2
HLS_{19}-NAV8	-2.749	6.961	0.138	0.89	agedico, agecat2
HLS_{19}-NAV9	4.387	64.875	<0.001*	1.39	
HLS_{19}-NAV10	-3.141	8.836	0.065	0.88	
HLS_{19}-NAV11	-0.946	2.247	0.690	0.99	
HLS_{19}-NAV12	0.662	5.624	0.229	1.08	pay bills*

*significant when sample size n = 720

Table S7 Item fit statistics of the HLS_{19}-NAV for Czech Republic (CAWI)

Item	Fit.res.	Chi sq<br>n = 1080	Chi sq p	Infit MNSQ	DIF<br>n=1080
HLS_{19}-NAV1	-1.962	3.883	0.422	0.99	
HLS_{19}-NAV2	-2.653	2.387	0.665	0.94	
HLS_{19}-NAV3	-0.206	3.580	0.466	1.07	
HLS_{19} -NAV4	-1.33	7.314	0.120	1.00	
HLS_{19} -NAV5	-4.23	8.569	0.073	0.90	
HLS_{19}-NAV6	-1.529	1.905	0.753	1.00	health#
HLS_{19}-NAV7	-0.837	4.046	0.400	1.05	gender*, agedico*, agecat2
HLS_{19}-NAV8	-3.163	4.620	0.329	0.92	
HLS_{19}-NAV9	-2.817	9.393	0.052	0.92	
HLS_{19}-NAV10	-4.711	14.941	0.005	0.86	
HLS_{19}-NAV11	-2.462	2.947	0.567	0.96	
HLS_{19}-NAV12	8.143	40.942	<0.001*	1.35	agedico, agecat2

non-uniform DIF

*significant when sample size n = 720

Table S8 Item fit statistics of the HLS19-NAV for Czech Republic (CATI)

Item	Fit.res.	Chi sq $n = 1080$	Chi sq p	Infit MNSQ	DIF n=1080
HLS19-NAV1	-0.634	2.344	0.673	1.04	
HLS19-NAV2	-1.712	4.686	0.321	0.98	
HLS19-NAV3	2.551	12.494	0.014	1.23	
HLS19 -NAV4	0.315	1.143	0.887	1.06	
HLS19 -NAV5	-2.825	10.762	0.029	0.90	
HLS19-NAV6	-1.378	10.145	0.038	1.01	
HLS19-NAV7	-0.033	6.388	0.172	1.04	
HLS19-NAV8	-1.487	1.175	0.882	1.00	gender*, agecat1*, soclevel*
HLS19-NAV9	-1.505	1.748	0.782	0.96	agedico*, agecat2*
HLS19-NAV10	-2.055	12.996	0.011	0.93	
HLS19-NAV11	-2.074	5.609	0.230	0.95	
HLS19-NAV12	0.494	5.882	0.208	1.13	

*significant when sample size $n = 532$

Table S9 Item fit statistics of the HLS19-NAV for France (CAWI)

Item	Fit.res.	Chi sq $n = 1080$	Chi sq p	Infit MNSQ	DIF n=1080
HLS19-NAV1	-0.449	5.691	0.223	1.05	
HLS19-NAV2	2.950	30.163	<0.001*	1.21	agedico, agecat, agecat2
HLS19-NAV3	-0.613	4.514	0.341	1.07	agedico*, agecat1 agecat2*, employment
HLS19 -NAV4	-3.251	14.361	0.006	0.95	
HLS19 -NAV5	-5.057	5.574	0.233	0.91	
HLS19-NAV6	-6.192	7.609	0.107	0.89	
HLS19-NAV7	-4.327	2.390	0.664	0.95	agedico*, agecat1*, agecat2*
HLS19-NAV8	-6.744	16.954	0.002	0.84	agedico*, agecat2*
HLS19-NAV9	8.595	133.836	<0.001*	1.54	gender
HLS19-NAV10	-6.894	13.873	0.008	0.84	
HLS19-NAV11	-4.236	3.309	0.508	0.94	
HLS19-NAV12	-2.100	9.500	0.050	1.00	

*significant when sample size $n = 720$

Table S10 Item fit statistics of the HLS$_{19}$-NAV for Germany (PAPI)

Item	Fit.res.	Chi sq $n = 1080$	Chi sq p	Infit MNSQ	DIF n=1080
HLS$_{19}$-NAV1	-0.817	4.704	0.319	0.97	
HLS$_{19}$-NAV2	-1.025	4.597	0.331	0.96	
HLS$_{19}$-NAV3	3.396	12.258	0.016	1.13	
HLS$_{19}$ -NAV4	-2.877	12.326	0.015	0.91	
HLS$_{19}$ -NAV5	-4.17	10.622	0.031	0.89	
HLS$_{19}$-NAV6	2.18	16.195	0.003	1.10	education
HLS$_{19}$-NAV7	-2.201	3.496	0.479	0.98	
HLS$_{19}$-NAV8	-0.684	2.859	0.582	1.02	
HLS$_{19}$-NAV9	1.876	9.821	0.044	1.10	
HLS$_{19}$-NAV10	-5.575	22.911	<0.001*	0.85	
HLS$_{19}$-NAV11	-0.823	0.303	0.990	1.00	
HLS$_{19}$-NAV12	3.099	10.659	0.031	1.13	

Table S11 Item fit statistics of the HLS$_{19}$-NAV for Portugal (CATI)

Item	Fit.res.	Chi sq $n = 1080$	Chi sq p	Infit MNSQ	DIF n=1080
HLS$_{19}$-NAV1	-3.567	0.31	0.989	1.05	
HLS$_{19}$-NAV2	-3.320	21.241	<0.001	1.02	education
HLS$_{19}$-NAV3	1.678	15.457	0.004	1.2	
HLS$_{19}$ -NAV4	-2.695	28.25	<0.001*	1.05	agecat1#, pay bills
HLS$_{19}$ -NAV5	-4.84	2.031	0.730	0.96	
HLS$_{19}$-NAV6	-5.247	11.959	0.018	0.96	pay bills*
HLS$_{19}$-NAV7	-6.114	20.112	0.001	0.86	agedico, agecat1
HLS$_{19}$-NAV8	-5.982	28.813	<0.001*	0.84	
HLS$_{19}$-NAV9	-3.65	33.106	<0.001*	1.09	pay bills
HLS$_{19}$-NAV10	-3.41	9.209	0.056	0.97	
HLS$_{19}$-NAV11	-4.267	10.961	0.027	0.99	
HLS$_{19}$-NAV12	-3.443	2.828	0.587	1.03	

non-uniform DIF

*significant when sample size $n = 720$

Table S12 Item fit statistics of the HLS_{19}-NAV for Slovenia (CAWI)

Item	Fit.res.	Chi sq *n* = 1080	Chi sq *p*	Infit MNSQ	DIF *n*=1080
HLS_{19}-NAV1	-1.660	17.662	0.001	1.0	-
HLS_{19}-NAV2	-2.834	14.727	0.005	0.96	-
HLS_{19}-NAV3	-2.553	1.626	0.804	0.96	-
HLS_{19} -NAV4	-3.101	5.204	0.267	0.95	-
HLS_{19} -NAV5	-5.688	10.606	0.031	0.86	-
HLS_{19}-NAV6	-3.364	2.433	0.657	0.95	-
HLS_{19}-NAV7	-4.145	8.528	0.074	0.93	-
HLS_{19}-NAV8	-2.724	21.827	<0.001	0.94	-
HLS_{19}-NAV9	-1.502	32.352	<0.001*	1.07	-
HLS_{19}-NAV10	-5.472	14.242	0.007	0.87	-
HLS_{19}-NAV11	0.332	2.027	0.731	1.1	-
HLS_{19}-NAV12	9.338	75.017	<0.001*	1.44	gender

*significant when sample size *n* = 720

Table S13 Item fit statistics of the HLS_{19}-NAV for Slovenia (CAPI)

Item	Fit.res.	Chi sq *n* = 1080	Chi sq *p*	Infit MNSQ	DIF *n*=1080
HLS_{19}-NAV1	-1.746	2.318	0.677	1.05	-
HLS_{19}-NAV2	-3.597	10.094	0.039	0.95	-
HLS_{19}-NAV3	-1.761	4.526	0.340	1.03	-
HLS_{19} -NAV4	2.183	33.614	<0.001*	1.17	-
HLS_{19} -NAV5	-7.081	11.787	0.019	0.87	-
HLS_{19}-NAV6	-3.536	2.433	0.657	1.0	-
HLS_{19}-NAV7	-6.574	7.148	0.128	0.87	-
HLS_{19}-NAV8	-3.315	15.39	0.004	0.96	-
HLS_{19}-NAV9	-3.671	28.511	<0.001*	1.02	-
HLS_{19}-NAV10	-8.211	11.998	0.017	0.82	-
HLS_{19}-NAV11	-3.332	3.028	0.553	1.01	-
HLS_{19}-NAV12	2.316	26.632	<0.001*	1.21	-

*significant when sample size *n* = 720

Table S14 Item fit statistics of the HLS$_{19}$-NAV for Switzerland (CAWI)

Item	Fit.res.	Chi sq $n = 1080$	Chi sq p	Infit MNSQ	DIF n=1080
HLS$_{19}$-NAV1	-1.502	3.269	0.514	1.01	
HLS$_{19}$-NAV2	-1.534	10.058	0.040	1.00	
HLS$_{19}$-NAV3	1.420	6.818	0.146	1.11	agedico*, agecat1*, agecat2*, employment*
HLS$_{19}$ -NAV4	-4.599	8.145	0.086	0.92	
HLS$_{19}$ -NAV5	-6.989	17.925	0.001	0.86	
HLS$_{19}$-NAV6	-4.693	2.181	0.702	0.94	
HLS$_{19}$-NAV7	-0.441	3.028	0.553	1.06	education*, pay bills*
HLS$_{19}$-NAV8	-2.371	3.511	0.476	0.98	agedico, education, pay bills
HLS$_{19}$-NAV9	2.711	42.022	<0.001*	1.22	gender, agedico, agecat2
HLS$_{19}$-NAV10	-6.540	11.014	0.026	0.86	
HLS$_{19}$-NAV11	-3.001	1.021	0.907	0.96	
HLS$_{19}$-NAV12	0.180	2.453	0.653	1.07	

*significant when sample size $n = 720$

Table S15 Item fit statistics of the HLS$_{19}$-NAV for Switzerland (CATI)

Item	Fit.res.	Chi sq $n = 1092$	Chi sq p	Infit MNSQ	DIF n=1080
HLS$_{19}$-NAV1	-0.888	1.422	0.840	0.98	-
HLS$_{19}$-NAV2	-0.298	6.894	0.142	1.08	-
HLS$_{19}$-NAV3	0.702	9.823	0.044	1.22	-
HLS$_{19}$ -NAV4	-1.570	2.996	0.559	0.86	-
HLS$_{19}$ -NAV5	-1.057	2.115	0.715	0.96	-
HLS$_{19}$-NAV6	-2.213	7.441	0.114	0.84	-
HLS$_{19}$-NAV7	-1.628	3.915	0.418	0.93	-
HLS$_{19}$-NAV8	-1.966	12.556	0.014	0.87	-
HLS$_{19}$-NAV9	1.552	25.594	<0.001*	1.31	health*
HLS$_{19}$-NAV10	-1.637	3.106	0.540	0.91	-
HLS$_{19}$-NAV11	-0.935	2.851	0.583	1.00	-
HLS$_{19}$-NAV12	-1.107	1.796	0.773	0.98	-

*significant when sample size $n = 192$

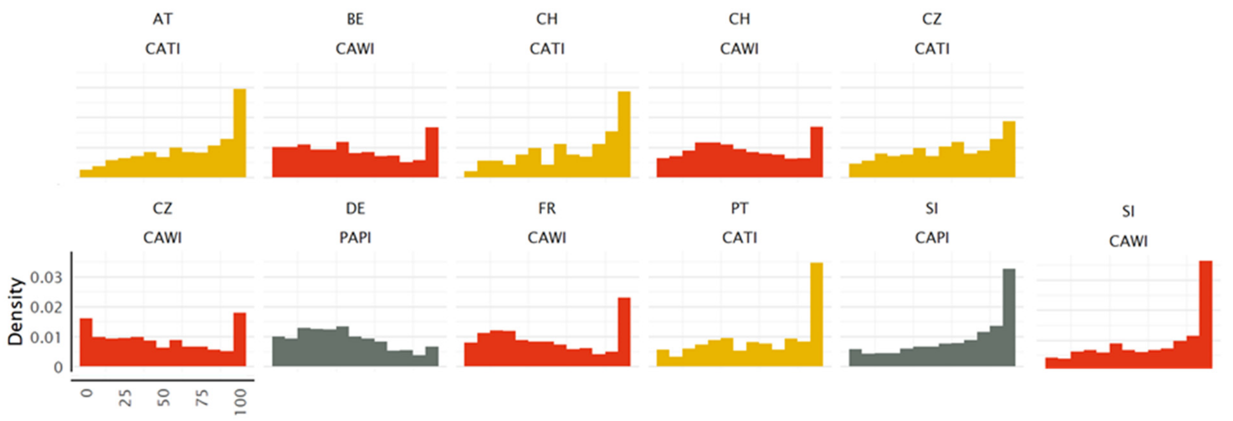

Figure S1. Distribution of the NAV-HL score (0-100) based on dichotomous data.

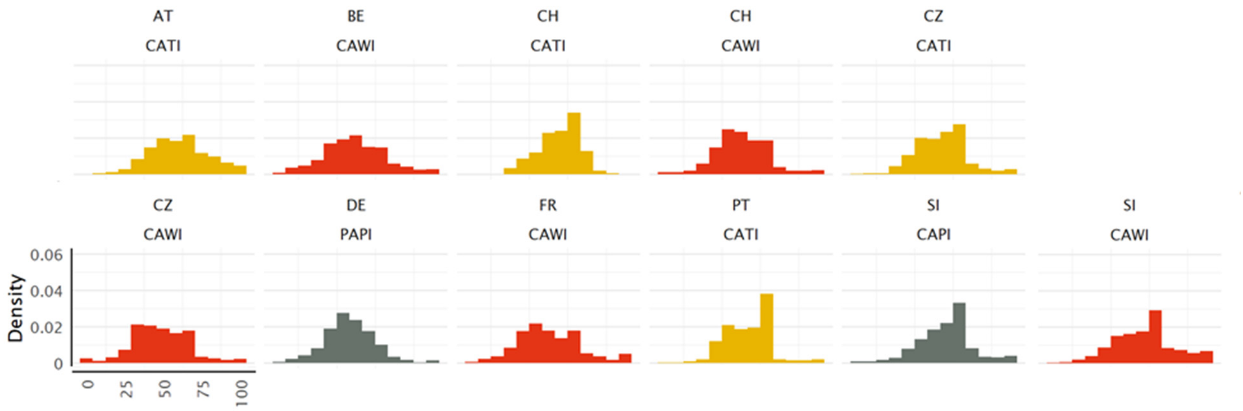

Figure S2. Distribution of the NAV-HL score (0-100) based on polytomous data.
